# Aberrant Expression of and Cell Death Induction by Engagement of the MHC-II Chaperone CD74 in Anaplastic Large Cell Lymphoma (ALCL)

**DOI:** 10.3390/cancers13195012

**Published:** 2021-10-07

**Authors:** Kathrin D. Wurster, Mariantonia Costanza, Stephan Kreher, Selina Glaser, Björn Lamprecht, Nikolai Schleussner, Ioannis Anagnostopoulos, Michael Hummel, Korinna Jöhrens, Harald Stein, Arturo Molina, Arjan Diepstra, Bernd Gillissen, Karl Köchert, Reiner Siebert, Olaf Merkel, Lukas Kenner, Martin Janz, Stephan Mathas

**Affiliations:** 1 Max-Delbrück-Center (MDC) for Molecular Medicine, 13125 Berlin, Germany; mariantonia.costanza@charite.de (M.C.); nikolai.schleussner@med.uni-heidelberg.de (N.S.); martin.janz@charite.de (M.J.); 2 Department of Hematology, Oncology and Cancer Immunology, Charité–Universitätsmedizin Berlin, corporate member of Freie Universität Berlin and Humboldt-Universität zu Berlin, 12200 Berlin, Germany; 3 Experimental and Clinical Research Center, a joint cooperation between the Charité and the MDC, 13125 Berlin, Germany; 4 Institute of Human Genetics, Ulm University, Ulm University Medical Center, 89081 Ulm, Germany; selina.glaser@uni-ulm.de (S.G.); reiner.siebert@uni-ulm.de (R.S.); 5 German Cancer Consortium (DKTK), German Cancer Research Center (DKFZ), 69120 Heidelberg, Germany; Michael.hummel@charite.de; 6 Institute of Pathology, Charité–Universitätsmedizin Berlin, 10117 Berlin, Germany; ioannis.anagnostopoulos@uni-wuerzburg.de (I.A.); korinna.joehrens@uniklinikum-dresden.de (K.J.); 7 Pathodiagnostik Berlin, 12099 Berlin, Germany; h.stein@pathodiagnostik.de; 8 Sutro Biopharma, South San Francisco, CA 94080, USA; amolina@sutrobio.com; 9 Department of Pathology and Medical Biology, University of Groningen, University Medical Centre Groningen, 9700 RB Groningen, The Netherlands; a.diepstra@umcg.nl; 10 Department of Hematology, Oncology, and Tumor Immunology, Charité–Universitätsmedizin Berlin, 13125 Berlin, Germany; bernhard.gillissen@charite.de; 11 Unit of Experimental and Laboratory Animal Pathology, Department of Pathology, Medical University of Vienna, 1090 Vienna, Austria; olaf.merkel@meduniwien.ac.at (O.M.); lukas.kenner@meduniwien.ac.at (L.K.); 12 European Research Initiative on ALK-related malignancies (ERIA), 1090 Vienna, Austria

**Keywords:** CD74, invariant chain, MHC-II, T cell lymphoma, *ALK* translocation

## Abstract

**Simple Summary:**

Anaplastic large cell lymphoma (ALCL) is a lymphoid malignancy considered to be derived from T cells. Currently, two types of systemic ALCL are distinguished: anaplastic lymphoma kinase (ALK)-positive and ALK-negative ALCL. Although ALK^+^ and ALK^−^ ALCL differ at the genomic and molecular levels, various key biological and molecular features are highly similar between both entities. We have developed the concept that both ALCL entities share a common principle of pathogenesis. In support of this concept, we here describe a common deregulation of CD74, which is usually not expressed in T cells, in ALCL. Ligation of CD74 induces cell death of ALCL cells in various conditions, and an anti-CD74-directed antibody-drug conjugate efficiently kills ALCL cell lines. Furthermore, we reveal expression of the proto-oncogene and known CD74 interaction partner MET in a fraction of ALCL cases. These data give insights into ALCL pathogenesis and might help to develop new treatment strategies for ALCL.

**Abstract:**

In 50–60% of cases, systemic anaplastic large cell lymphoma (ALCL) is characterized by the t(2;5)(p23;q35) or one of its variants, considered to be causative for anaplastic lymphoma kinase (ALK)-positive (ALK^+^) ALCL. Key pathogenic events in ALK-negative (ALK^−^) ALCL are less well defined. We have previously shown that deregulation of oncogenic genes surrounding the chromosomal breakpoints on 2p and 5q is a unifying feature of both ALK^+^ and ALK^−^ ALCL and predisposes for occurrence of t(2;5). Here, we report that the invariant chain of the MHC-II complex CD74 or li, which is encoded on 5q32, can act as signaling molecule, and whose expression in lymphoid cells is usually restricted to B cells, is aberrantly expressed in T cell-derived ALCL. Accordingly, ALCL shows an altered DNA methylation pattern of the *CD74* locus compared to benign T cells. Functionally, CD74 ligation induces cell death of ALCL cells. Furthermore, CD74 engagement enhances the cytotoxic effects of conventional chemotherapeutics in ALCL cell lines, as well as the action of the ALK-inhibitor crizotinib in ALK^+^ ALCL or of CD95 death-receptor signaling in ALK^−^ ALCL. Additionally, a subset of ALCL cases expresses the proto-oncogene MET, which can form signaling complexes together with CD74. Finally, we demonstrate that the CD74-targeting antibody-drug conjugate STRO-001 efficiently and specifically kills CD74-positive ALCL cell lines in vitro. Taken together, these findings enabled us to demonstrate aberrant CD74-expression in ALCL cells, which might serve as tool for the development of new treatment strategies for this lymphoma entity.

## 1. Introduction

Systemic anaplastic large cell lymphoma (ALCL), a subgroup of peripheral T cell lymphomas (PTCL), is characterized by large atypical cells and expression of the TNF receptor family member CD30 [1,2,3]. Among systemic ALCLs, the current 2016 WHO classification distinguishes two entities: anaplastic lymphoma kinase (ALK)-positive (ALK^+^) ALCL, which is in most cases characterized by the t(2;5)(p23;q35) resulting in the expression of the oncogenic NPM-ALK fusion protein and represents approx. 50–60% of all ALCL cases; and ALK-negative (ALK^−^) ALCL, which lacks t(2;5) and ALK expression [4]. Despite recent progress [5,6], key pathogenic events in ALK^−^ ALCL are still less clarified. Although phenotypically highly similar [1], both ALCL entities show differences with respect to genomic aberrations, gene expression profile [7,8,9], miRNA expression pattern [10] and prognosis, which is unfavorable in ALK^−^ ALCL [11]. Given the rather poor prognosis of subgroups of ALK^−^ ALCL patients and of refractory or relapsed ALCL patients, alternative treatment strategies are required in particular for this group of patients.

Despite differences at the genomic and molecular levels, ALK^+^ and ALK^−^ ALCL share various key biological and molecular features [7,12,13,14,15,16,17]. We have previously developed the concept that deregulation of genes surrounding the ALCL-characteristic putative breakpoints on chromosomes 2p and 5q might be similarly important for ALCL lymphomagenesis as the resulting translocation itself [14,15,18]. In this context, we identified an ALCL-specific deregulation of breakpoint-surrounding genes with transforming capacity, including the oncogenic tyrosine-kinase receptor *CSF1R* or the activator protein-1 (AP-1) family member Fos-related antigen 2 (*FRA2*) [14].

In an extension of said work, we demonstrate here in ALCL an unexpected expression of CD74 (encoded on 5q32; also known as the invariant chain of major histocompatibility complex [MHC]-II, li), regardless of t(2;5). CD74 belongs to the family of single-pass type II membrane proteins, serves as chaperone for MHC-II molecules in antigen-presenting cells and occupies the MHC-II binding groove to prevent inappropriate binding of peptides. To allow for antigen presentation, CD74 is degraded, and its fragment class-II-associated invariant chain peptide (CLIP) remains bound to the MHC-II binding groove, which is ultimately removed to permit peptide binding and subsequent antigen presentation (for a recent review, see [19]). Initially thought to show an expression pattern largely restricted to B cells and to be localized intracellularly [20,21,22], CD74 expression has been demonstrated to appear on the cell surface and to be present on antigen-presenting cells, such as macrophages or dendritic cells, as well as certain solid malignancies including breast and gastrointestinal cancers [19,21,23,24,25]. In contrast to B cells, T cells usually lack CD74 expression, and CD74 has previously been reported only on cutaneous T cell-derived lymphoma cell lines as well as certain T cell subsets following activation [21,24,26,27]. Only recently, CD74 expression has been analyzed on a broad lymphoma panel including B and T cell lymphoma subtypes [28]. Apart from its function as an MHC-II chaperone, CD74 can act as a signaling molecule, forming receptor complexes with, e.g., CD44, chemokine (C-X-C motif) receptor 4 (CXCR4) and the proto-oncogene MET [29,30,31,32,33]. Upon activation of CD74 by binding of its ligand, macrophage migration-inhibitory factor (MIF), or by antibody stimulation, signaling is activated, resulting either in enhanced survival and proliferation or induction of growth arrest and cell death depending on the cellular context [34,35,36,37]. CD74 activation thereby interferes with NF-κB, MAPK, Akt and MET activation [32,33,35,38].

The consistent expression of CD74 on normal and transformed B cells, its rapid internalization, as well as growth-inhibitory and death-inducing effects in vitro on B cell non-Hodgkin lymphoma (B-NHL) cells, brought CD74 into focus as a therapeutic target [39]. Therefore, unconjugated and drug-conjugated monoclonal anti-CD74-antibodies are currently being investigated in clinical trials for the treatment of B-NHL [40,41,42]. We here describe expression of CD74 on T cell-derived ALCL and show that targeting of CD74 strongly affects the viability of ALCL cell lines, suggesting that CD74 serves as a therapeutic target in ALCL.

## 2. Materials and Methods

### 2.1. Cell Lines and Culture Conditions

ALCL (Karpas 299 (in the following referred to as K299), SU-DHL-1, DEL, JB6, all ALK^+^; Mac-1, Mac-2A, FE-PD, DL40, all ALK^−^) and T cell leukemia-derived (Jurkat, KE-37, Molt-14, H9) cell lines, the classical Hodgkin lymphoma-derived cell line L428, as well as the pro-B lymphoblastic leukemia cell line Reh, were cultured as previously described [14]. Cell line identity was confirmed by short tandem repeat (STR) analysis. Where indicated, cells were cultured in the presence of sodium azide-free anti-CD74 antibody (sc20062L; Santa Cruz Biotechnology, Heidelberg, Germany), the respective IgG_1_ isotype control (IC; sc-3877L; Santa Cruz), IgG(Fc)-specific F(ab′)_2_-fragments (115-006-071; Dianova, Hamburg, Germany), the chemotherapeutics vincristine or etoposide (both Calbiochem, Darmstadt, Germany), the ALK-inhibitor crizotinib (PF-02341066, kindly provided by Pfizer, San Diego, CA, USA) or the respective DMSO control (Carl Roth, Karlsruhe, Germany), the agonistic anti-CD95 antibody CH-11 (IM1504; Coulter-Immunotech, Krefeld, Germany) or the respective IgM control (290-010; Biomol, Hamburg, Germany), or the MET inhibitors Foretinib (S1111; Selleckchem, München, Germany) and JNJ-38877605 (S1114; Selleckchem) at the indicated concentrations. In addition, cells were treated with the CD74-targeting ADC STRO-001 or, as a control, ADC GFP-SC236 targeting GFP (both from Sutro Biopharma) at the indicated concentrations. The LD_50_ was determined using non-linear regression in Prism v8. Peripheral mononuclear cells were purified from peripheral blood of healthy donors according to standard protocols. The use of human material was approved by the Local Ethics Committee of the Charité–Universitätsmedizin Berlin, and performed in accordance with the Declaration of Helsinki.

### 2.2. RNA Preparation and RT-PCR Analyses

Total RNA preparation was performed as previously described [14]. For RT-PCR analyses, cDNA synthesis was performed with the 1st Strand cDNA Synthesis Kit (AMV; Roche Diagnostics, Mannheim, Germany). Semi-quantitative RT-PCR analyses were performed as described. Primers used for RT-PCR analyses were as follows: *CD74* sense (s) 5′-GACAGTCACCTCCCAGAACC, *CD74* antisense (as) 5′-GGCAGATAGTTGCCGTTCTC; *GAPDH* s 5′-ATGCTGGCGCTGAGTAC, *GAPDH* as 5´-TGAGTCCTTCCACGATAC; *MET* s 5′-ATCGATCTGCCATGTGTGC, *MET* as 5′-CACATATGGTCAGCCTTGTCC; *CD44* s 5′-AATATAACCTGCCGCTTTGC, *CD44* as 5′-CAGGTCTCAAATCCGATGCT; *MIF* s 5′-CCGAGAAGTCAGGCACGTAG, *MIF* as 5′-ATAGTTGATGTAGACCCTGTCCG; *CXCR4* s 5′-GCCGACCTCCTCTTTGTCAT, *CXCR4* as 5′-TAGTAAGGCAGCCAACAGGC. All PCR products were verified by sequencing.

### 2.3. Immunofluorescence and Flow Cytometry, Analysis of Apoptosis and Light Microscopy

For the analysis of CD74 cell surface expression, cells were incubated with monoclonal antibody to CD74 (sc-20062 or sc-6262; both from Santa Cruz), monoclonal antibody to MET (MAB3582; R&D Systems, Wiesbaden, Germany) or the respective isotype control (MAB002; R&D Systems), followed by incubation with a phytoerythrin (PE)-conjugated F(ab′)_2_ fragment (115-116-071; Dianova). For the analysis of primary lymphoid cells, indirect staining for CD74 was performed in a first step as described above, followed by incubation with APC-labeled anti-CD19 (C7224; Dako, Hamburg, Germany) or anti-CD4 (IM2468; Beckman Coulter, Krefeld, Germany) antibodies. Immunofluorescence was analyzed using a FACSAria flow cytometer and CELLQuest software (Becton Dickinson). The percentage of viable and apoptotic cells was determined by Annexin V-FITC/propidium iodide (PI) double staining (Bender MedSystems) and flow cytometry using a FACSAria flow cytometer. Cells double negative for Annexin V-FITC and PI were considered as viable cells. For light microscopy, a Leica CTR6000 microscope equipped with a Leica DFC350FX camera was used.

### 2.4. Western Blot Analyses

Whole-cell extract preparation and Western blot analyses were performed as previously described [14]. For Western blot analyses, 30 μg of whole cell extracts were used. The following primary antibodies were used: anti-CD74 (sc-20062 and sc-6262; both Santa Cruz), anti-MET (D1C2; Cell Signaling Technology, Frankfurt (Main), Germany), anti-poly[ADP-ribose](PARP)-1 (#9542; Cell Signaling Technology), cleaved PARP (Asp214) (#9541, Cell Signaling Technology), anti-β-actin (A5316; Sigma-Aldrich, Taufkirchen, Germany). Membranes were incubated with horseradish peroxidase-conjugated secondary antibodies. Bands were visualized using an enhanced chemiluminescence system (Amersham Pharmacia Biotech, Freiburg, Germany).

### 2.5. Immunohistochemistry (IHC)

The detection of CD74 protein in formalin-fixed and paraffin-embedded tissue sections was performed employing the anti-CD74 antibodies sc-20062 and sc-6262 (both Santa Cruz) at a dilution of 1:1000 after a 20 min treatment in EDTA buffer (Retrieval solution 2; EDTA-buffer pH 8.8 at 98 °C for 20 min). Bound antibody was visualized using the alkaline phosphatase anti-alkaline phosphatase method and FastRed as chromogen (DAKO) or, after peroxidase blocking, DAB-chromogen. For c-MET detection, the prediluted anti-total c-Met (clone SP44) rabbit monoclonal antibody was obtained from Ventana (Ventana Medical System). Immunostaining was carried out according to the manufacturer´s protocol on the BenchMark Ultra platform from Ventana utilizing the ultraView detection kit.

### 2.6. DNA Methylation Analyses Using Illumina Infinium Arrays

DNA methylation measurements were performed after modification with the EZ DNA Methylation kit (ZymoResearch, Irvine, CA, USA) using either Infinium^®^ HumanMethylation450 (450K) or Infinium^®^ MethylationEPIC (EPIC) BeadChips (Illumina Inc., San Diego, CA, USA). Moreover, published and publicly available array-based DNA methylation data were mined (see Supplementary Materials). DNA methylation values from primary ALCL were generated in-house or obtained from Hassler et al. [16]. In addition, ALK positive (SU-DHL-1, K299, JB6) and negative (FE-PD, Mac-2A) ALCL cell lines as well as T-ALL (CCRF-CEM, Jurkat, MOLT-3 and MOLT-4) cell lines were investigated (all obtained from DSMZ, Germany). Beside neoplastic samples, the study cohort was complemented with publicly available data from various cell populations covering T- and B-lineage differentiation as well as from monocytes and macrophages (for references, see Supplementary Materials). Included cell types were hematopoietic stem cells, pre B-cells, immature B-cells, naïve B-cells, tonsillar naïve B-cells, germinal center founder cells, germinal center derived B-cells, early non class-switched B-cell, non class-switched memory B-cells, class-switched memory B-cells, splenic marginal zone B-cells, tonsillar plasma cells, bone marrow plasma B-cells and various T cell subpopulations. For the analyses, raw idat files were processed using the minfi package within the R statistical program (www.R-project.org, accessed on 31 August 2020). Subsequently, beta values were calculated representing the percentage of DNA methylation at a certain cytosine base. For downstream analyses, rs loci, loci on gonosomes and loci with a detection *p* value of >0.01 were excluded from further analysis. Finally, 15 CpG loci mapping to the *CD74* gene locus were extracted from the dataset and visualized as a heatmap using the OMICS Explorer 3.6 (Qlucore; Lund, Sweden).

### 2.7. Statistics

Statistical analyses were performed in R v2.9.1 (http://www.r-project.org/, accessed on 23 September 2021) and graphPad Prism Version 5.0. For statistical analyses of cell death induction, numbers of viable cells per sample were fitted using a generalized linear, negative binominal model with a log-link function. For experiments with combinatorial treatments, all treatments were specified as factors with respective number of factor levels. For every treatment combination, one main-effect and one interaction-effect model were calculated. *p* values were adjusted for multiple testing using Tukey’s honestly significant difference test.

## 3. Results

### 3.1. Aberrant CD74 Expression in ALCL Cell Lines

**Figure 1:**
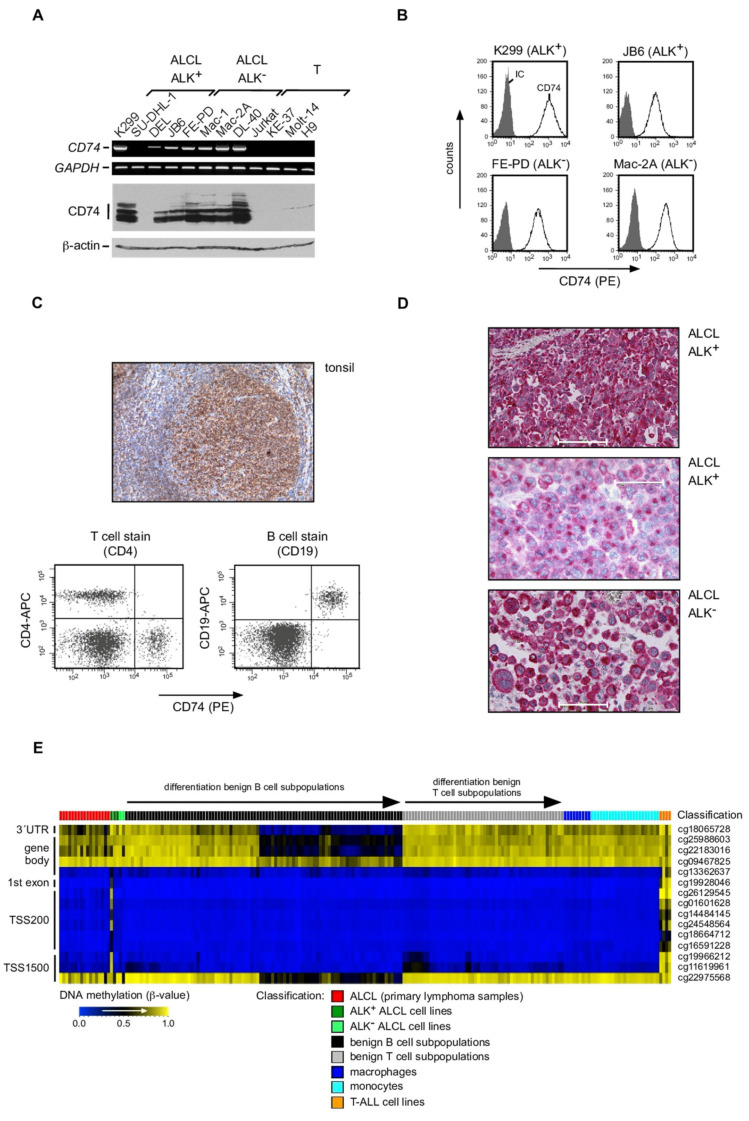
CD74 expression in ALCL. (**A**) Analysis of CD74 mRNA (upper panel) and protein (lower panel; antibody sc-6262) expression in various ALK^+^ and ALK^−^ ALCL as well as T cell control cell lines, as indicated. The expression levels of *GAPDH* and of β-actin were analyzed as controls, respectively. Note, that various CD74 protein bands of different sizes are detectable. (**B**) Cell surface expression analysis of CD74 in two ALK^+^ (K299, JB6) and two ALK^−^ (FE-PD, Mac-2A) ALCL cell lines by extracellular flow cytometry (antibody sc-20062). Open histogram, CD74 staining; filled histogram, isotype control (IC). (**C**) Physiological CD74 protein expression in the human B cell compartment. Upper panel, CD74 immunohistochemistry (IHC) in normal human tonsillar tissue. Note that the B cell compartment including the germinal center strongly stains for CD74 (brown signal), whereas the T cell compartment lacks CD74 expression. Lower panels, expression analyses of CD74 on T and B cells of human peripheral blood. Left, CD74 and CD4 double-staining of human peripheral blood mononuclear cells (PMNC). Note, that CD4-positive cells (upper left quadrant) do not stain for CD74. Right, CD74 and CD19 double-staining of human PMNC of peripheral blood. Note that CD19-positive cells do express CD74 (upper right quadrant). (**D**) Representative CD74 IHC analyses of two primary human ALK^+^ (upper panel, strong staining; center, weak staining with prominent staining of the Golgi area) cases and one ALK^−^ (lower panel) ALCL case. CD74 signal is in red. Scale bars: upper panel 100 μm, center and lower panel 50 μm. (**E**) Heatmap showing DNA methylation levels at 15 CpG loci associated with the gene *CD74* in primary ALCL samples (*n* = 16) and cell lines (SU-DHL-1, K299, JB6, FE-PD, Mac-2A), T-ALL cell lines (CCRF-CEM, Jurkat, MOLT 3 and MOLT 4), monocytes (*n* = 23), and macrophages (*n* = 9) as well as various non-malignant T- and B-cell subpopulations. Columns represent samples and rows depict loci. The loci are ordered according to their genomic position.

Analyzing the expression of genes that are located in the same chromosome regions of the putative t(2;5)(p23;q35) breakpoints in ALCL, we identified in ALCL an aberrant expression of the *CD74* gene (Figure 1) that is located on 5q32. To this end, we used a panel of ALK^+^ and ALK^−^ ALCL cell lines and non-ALCL T cell-derived control cell lines already used in our previous studies [13,14,15,17]. Robust *CD74* mRNA expression was observed in all ALCL cell lines independent of their ALK expression status, except for SU-DHL-1 cells, whereas it was absent in the T cell control cell lines (Figure 1A, upper panel; Figure S2). Using different CD74 antibodies, we confirmed strong CD74 protein expression in ALCL cell lines by immunoblotting (Figure 1A, lower panel; Figures S1A and S2), demonstrating multiple bands that most likely reflect the expression of different splice variants and glycosylation levels [19]. In antigen-presenting cells, CD74 is often found intracellularly with low expression levels on the cell surface due to rapid internalization and intracellular degradation [39]. However, in all ALCL cell lines with CD74 expression (see Figures S1A and S2), we observed robust CD74 protein expression on the cell surface by flow cytometry (Figure 1B and Figure S1B).

### 3.2. CD74 Expression in Human Lymphocytes and Primary ALCL Cases

To confirm our cell line data, we analyzed lymphoid cells from peripheral blood of healthy donors, normal lymphoid tissue and primary ALCL samples by flow cytometry and immunohistochemistry (IHC; Figure 1C,D and Figure S1C, and Table 1). Our analyses confirmed the restriction of CD74 expression, among lymphoid cells, to the B cell compartment. Thus, in tissues of non-neoplastic human palatine tonsils, only B cells (and in accordance with the known expression pattern, macrophages and dendritic cells) stained positive, whereas T cells were negative for CD74 (Figure 1C, upper panel). Similarly, in the analysis of CD74 expression by extracellular flow cytometry on mononuclear cells from peripheral blood of healthy donors, only CD19^+^ B cells were positive for CD74, whereas CD4 T cells (the putative cellular compartment of ALCL origin) lacked CD74 expression (Figure 1C, lower panel; Figure S1C). Next, we evaluated a series of 35 primary human ALK^+^ and 21 ALK^−^ ALCL cases for CD74 expression by IHC (Figure 1D and Table 1). These analyses confirmed CD74 expression, albeit at different expression levels and frequently with a predominantly cytoplasmic staining, in all ALCL cases analyzed, independent of ALK expression (Table 1). In summary, these data demonstrate that CD74 is aberrantly expressed in, and is a unifying feature of, ALK^+^ and ALK^−^ ALCL.

### 3.3. DNA Methylation Analyses of the CD74 Locus in ALCL

In order to investigate whether altered DNA methylation could affect expression of *CD74* in ALCL, array-based DNA methylation data of primary ALCL samples and ALCL cell lines were mined, and the DNA methylation levels of 15 CpGs at the *CD74* locus were determined (Figure 1E). Findings were compared to those in various B- and T-cell subpopulations, monocytes, macrophages and T-ALL cell lines as well (Figure 1E). In contrast to all controls, we observed a strong DNA methylation around the transcription start site (TSS) in the T-ALL cell lines and in SU-DHL-1. Several loci associated with the 3′UTR, parts of the gene body and 5′ of the gene (TSS1500) were generally strongly methylated, but lost DNA methylation during the progression of B-cell differentiation, in line with the restricted CD74 expression pattern in mature B-cells [20,21,22,43]. Notably, the same CpGs showed decreased DNA methylation in several primary ALCL samples and ALCL cell lines compared to normal T-cell subsets, further supporting unusual CD74 expression in this putative T cell-derived lymphoma entity.

### 3.4. Induction of Apoptosis in ALCL Following CD74 Ligation

**Figure 2:**
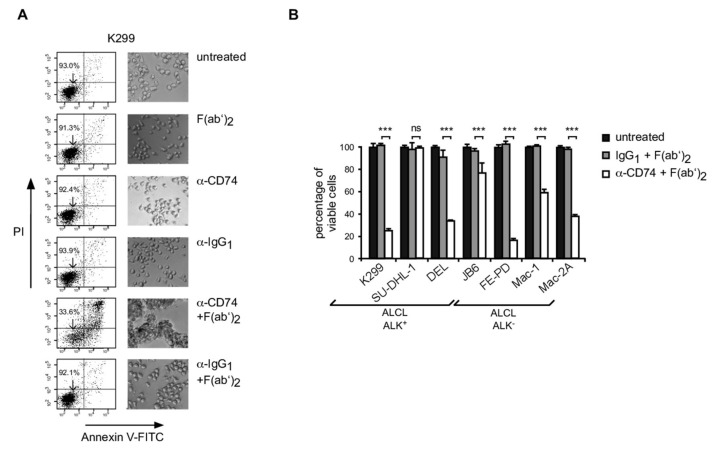
Induction of apoptosis of ALCL cell lines following CD74 ligation. (**A**) K299 cells were left untreated, or treated for 48 h with 20 μg F(ab′)_2_-fragments (F(ab′)_2_), 5 μg anti-CD74 (α-CD74) or the respective IgG_1_ isotype control (α-IgG_1_), or anti-CD74 or anti-IgG_1_ in combination with F(ab′)_2_-fragments, as indicated. Left, flow cytometry analyses of the respectively treated cells following Annexin V-FITC and PI-staining. The percentage of viable, Annexin V-FITC/propidium iodide (PI) double-negative cells is indicated. Right, transmitted-light microscopy of the cells. Note that strong clustering of cells is observed following treatment with anti-CD74 in combination with F(ab′)_2_. One of three independent experiments is shown. (**B**) Various ALK^+^ and ALK^−^ cell lines were left untreated (black columns), or were treated for 72 h with anti-CD74 in combination with F(ab′)_2_ (open columns), or, as control, anti-IgG_1_ in combination with F(ab′)_2_ (grey columns) as described in (A). Note that SU-DHL-1 cells, which lack CD74 expression, do not respond to CD74 ligation. One of four independent experiments is shown. Error bars denote SDs. ***, *p* < 0.001. ns, not significant.

CD74 is currently being explored as a therapeutic target particularly in B-NHL [40,41,42]. We reasoned that CD74 might also be a suitable target structure in ALCL. To this end, we explored the effects of CD74 ligation on the viability of ALCL cell lines (Figure 2 and Figure S1D). We cultured various ALCL cell lines in the presence of a monoclonal anti-CD74 or IgG control antibody without or, to enforce crosslinking and to mimic the presence of Fcγ-receptor-bearing cells, with Fcγ-specific F(ab′)_2_-fragments (Figure 2A,B). We measured induction of cell death by Annexin V-FITC/PI staining and subsequent flow cytometry at various times (24 h, 48 h and 72 h). Cell clustering was observed in samples treated with anti-CD74 in combination with F(ab′)_2_-fragment as documented by light microscopy (Figure 2A, right panels). Importantly, in CD74^+^ ALCL cell lines, we observed a time- and dose-dependent induction of apoptosis (Figure 2A, left panel, and Figure 2B; Figure S1D). Although the extent of cell death varied between ALCL cell lines, all CD74 positive cell lines showed a significant degree of apoptotic cell death (Figure 2B). In contrast, SU-DHL-1 cells, which do not express CD74 (see Figure 1A; Figure S2), were not affected by anti-CD74 treatment (Figure 2B).

### 3.5. CD74 Ligation Sensitizes ALCL Cell Lines for Various Apoptosis-Inducing Agents

**Figure 3:**
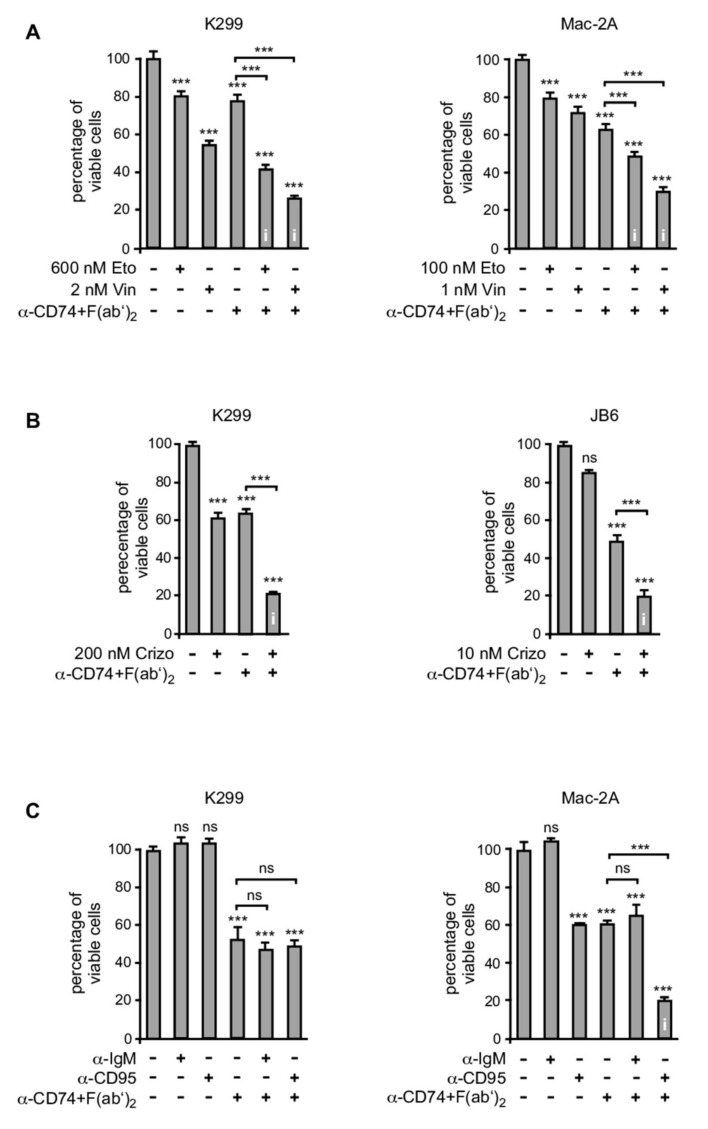
CD74 ligation sensitizes ALCL cell lines for various apoptosis-inducing stimuli. (**A**) Increased induction of apoptosis following CD74 ligation in combination with conventional chemotherapeutics. K299 and Mac-2A cells were left untreated, or were treated for 48 h with etoposide (Eto), vincristine (Vin), anti-CD74 in combination with F(ab′)_2_ (α-CD74+F(ab′)_2_; K299 with 2 μg F(ab′)_2_ and 0.5 μg anti-CD74, Mac-2A with 8 μg F(ab′)_2_ and 2 μg anti-CD74) or the respective combinations, as indicated. In all panels of this figure, the percentage of viable cells (Annexin V-FITC and PI double negative) is shown following Annexin V-FITC/PI staining and flow cytometry. Note that significant interaction effects (indicated by an ‘i’ in the respective columns) are observed in both cell lines by the combined treatment with chemotherapeutics and crosslinked α-CD74. (**B**) Increased induction of apoptosis following CD74 ligation and concomitant treatment with the ALK-inhibitor crizotinib. The ALK^+^ cell lines K299 and JB6 were left untreated or were treated for 24 h (K299) or 48 h (JB6) with crizotinib (crizo), anti-CD74 in combination with F(ab′)_2_ (α-CD74+F(ab′)_2_; K299 with 4 μg F(ab′)_2_ and 1 μg anti-CD74, JB6 with 10 μg F(ab′)_2_ and 2.5 μg anti-CD74), or crizotinib together with crosslinked anti-CD74, as indicated. Note that in both cell lines, significant interaction effects are observed by treatment with crizotinib in combination with crosslinked α-CD74. (**C**) Increased induction of apoptosis following CD74 ligation in combination with anti-CD95 in ALK^–^ ALCL cells. The ALK^+^ cell line K299 and the ALK^–^ cell line Mac-2A were left untreated, or were treated for 40 h with an agonistic CD95 antibody (α-CD95; K299 500 ng/mL; Mac-2A 50 ng/mL) or the respective isotype control (α-IgM), anti-CD74 in combination with F(ab′)_2_ (α-CD74+F(ab′)_2_; K299 with 4 μg F(ab′)_2_ and 1 μg anti-CD74, Mac-2A with 8 μg F(ab′)_2_ and 2 μg anti-CD74), or the respective combinations, as indicated. Note that a significant interaction effect is observed in Mac-2A but not in K299 cells. Error bars denote SDs. ***, *p* < 0.001. ns, not significant. i, significant interaction effect. For each experimental approach, one of three independent experiments is shown.

We next investigated whether CD74 ligation sensitizes ALCL cells for induction of cell death by other pharmacological agents or death-receptor engagement (Figure 3). First, we explored the combined treatment of cross-linked anti-CD74 and conventional chemotherapeutics with respect to a synergistic induction of cell death (Figure 3A). Here, we focused on etoposide and vincristine, which are both used for the treatment of ALCL [2]. ALK^+^ K299 and ALK^−^ Mac-2A cells were treated either with each chemotherapeutic, or crosslinked anti-CD74 alone using concentrations that only moderately induce cell death, or the respective combinations (Figure 3A). In these experiments, the combined treatment with each of the chemotherapeutics with anti-CD74 led to enhanced cell death induction (Figure 3A).

Second, we analyzed the effects of a combined treatment of the CD74 antibody with the ALK-inhibitor crizotinib. The latter has been successfully used for the treatment of ALK^+^ non-small cell lung cancer (NSCLC) [44] and is an effective treatment option for ALK^+^ ALCL patients [45]. To this end, the ALK^+^ cell lines K299 and JB6 were treated with each agent alone at concentrations that induced approx. 40% cell death, or with both agents in combination (Figure 3B). Combined treatment resulted in a substantial synergistic induction of cell death of around 80 % of cells (Figure 3B).

Third, we observed that a combined treatment of crosslinked anti-CD74 with an agonistic CD95 antibody led to a synergistic increase in the induction of apoptosis in ALK^–^ Mac-2A cells, while each substance alone induced cell death only in a moderate fashion under the conditions applied (Figure 3C, right panel). In ALK^+^ ALCL cell lines, treatment with anti-CD95 had no effect on cell viability and did not enhance cell death induction by crosslinked anti-CD74 antibody (Figure 3C, left panel).

### 3.6. Expression of MET in ALCL

**Figure 4:**
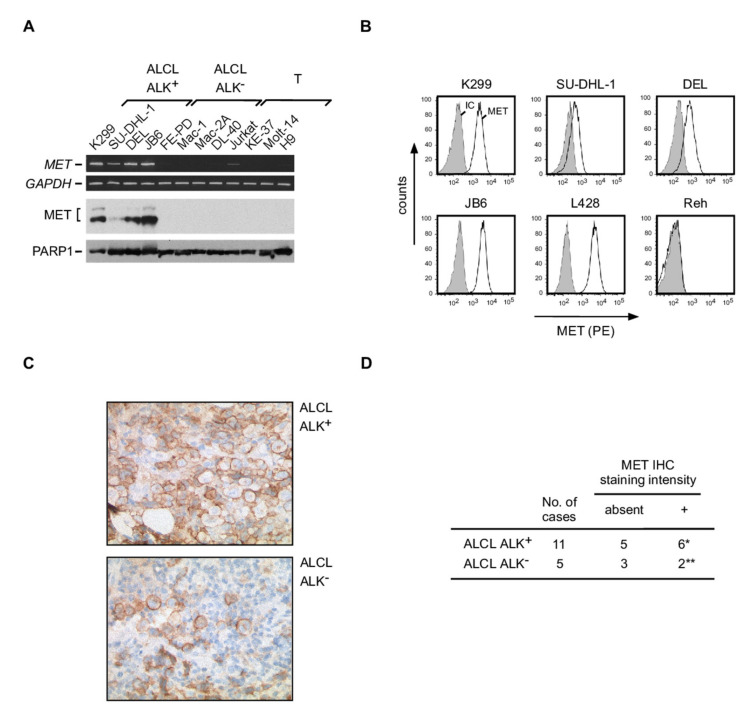
Expression of MET in ALCL. (**A**) Analysis of MET mRNA (upper panel) and protein (lower panel) expression in various ALK^+^ and ALK^–^ ALCL as well as T cell control cell lines, as indicated. The expression levels of *GAPDH* and of PARP-1 were analyzed as controls, respectively. Note that two MET bands are detectable at the protein level, corresponding to the precursor protein pro-MET (upper band) as well as MET (lower band). (**B**) Cell surface expression analysis of MET in four ALK^+^ ALCL cell lines (K299, SU-DHL-1, DEL, JB6) by extracellular flow cytometry. L428 cells with a known MET expression [48] were included as positive control, Reh cells as negative control. Open histogram, MET staining; filled histogram, isotype control (IC). (**C**) Examples of MET IHC of an ALK^+^ ALCL case (upper panel) and an ALK^–^ ALCL case (lower panel) with moderate tumor cell positivity. Note the cytoplasmic and partially membranous staining of the large ALCL cells. Magnification, 40X. (**D**) Results of MET IHC analyses in ALK^+^ and ALK^−^ ALCL. *, the percentage of positive ALCL cells among the cases was as follows: in 2 cases 60% of the lymphoma cells stained positive (in both cases including membranous staining), in 1 case 10%, in 2 cases 5%, in 1 case single positive cells. **, the percentage of positive ALCL cells among the cases was as follows: in 1 case 40%, in 1 case 10%. Staining intensity is indicated as follows: +, weak.

A number of CD74 interaction partners involved in CD74 signaling have been described, including CD44, the CD74 ligand MIF, the oncogenic tyrosine-kinases receptor MET and the C-X-C motif chemokine receptor CXCR4 [29,31,33,46]. Expression analyses in our cell line panel demonstrated that all putative interaction partners are expressed at least in a subset of ALCL cell lines (Figure 4 and Figure S1E). For further analysis, we focused on the receptor tyrosine kinase MET given that it has high oncogenic potential and can be targeted by small molecule inhibitors already in clinical use [47] (Figure 4). Interestingly, MET mRNA (Figure 4A, upper panel; Figure S2) and protein (Figure 4A, lower panel; Figure S2) expression analyses revealed a robust MET expression in all ALK^+^ ALCL cell lines, whereas ALK^−^ ALCL cell lines lacked MET expression. In addition, MET expression on the cell surface of ALK^+^ ALCL cell lines was confirmed by flow cytometry (Figure 4B). MET immunohistochemistry on 11 ALK^+^ and 5 ALK^–^ ALCL primary tissue samples (Figure 4C,D) revealed MET positivity, although moderate, in 6 out of 11 ALK^+^ and 2 out of 5 ALK^–^ ALCL cases. Direct interactions between CD74 and MET have previously been described in other cell types [33]. However, despite various immunoprecipitation approaches, we were unable to detect direct interactions of these proteins in ALCL cell lines. Functionally, we tested the effect of the MET inhibitors Foretinib as well as JNJ-38877605 on the ALK^+^ ALCL cell lines K299 and JB6, both with MET expression, and on the ALK^−^ ALCL cell line FE-PD and the T control cell line Jurkat, both the latter without MET expression, for induction of cell death. Both inhibitors did not alter viability of these cell lines.

### 3.7. The CD74-Targeting Antibody-Drug Conjugate STRO-001 Efficiently Kills ALCL Cell Lines

**Figure 5:**
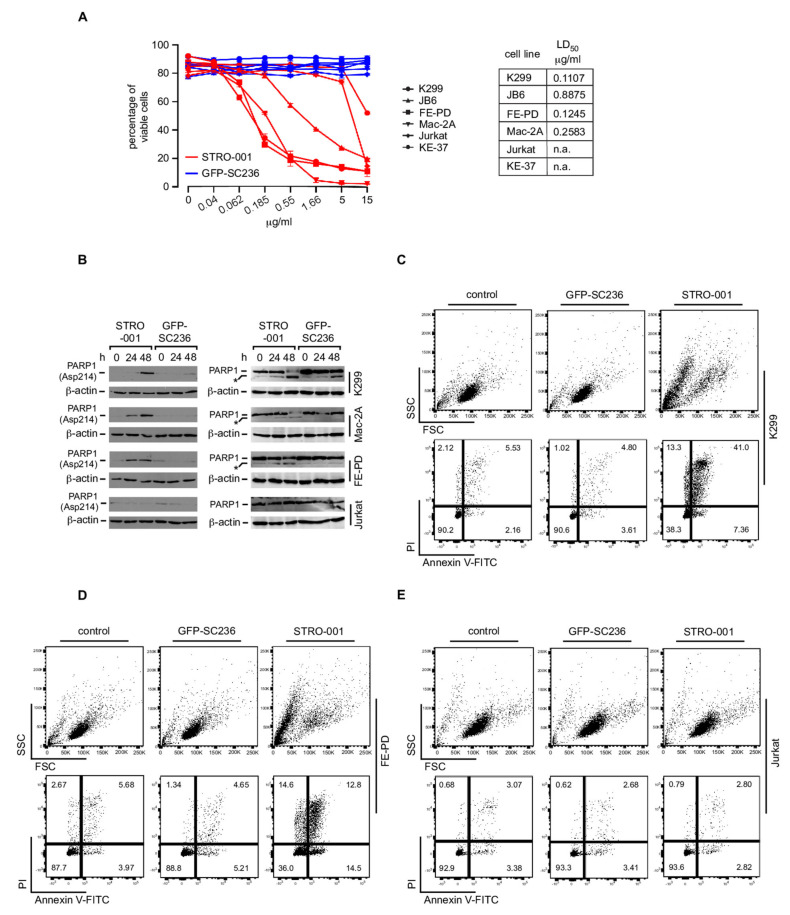
Induction of apoptotic cell death by the CD74-targeting antibody-drug conjugate (ADC) STR-001. (**A**) Left, the indicated ALCL cell lines (K299, JB6, FE-PD, Mac-2; all CD74 positive) as well as the T cell control cell lines Jurkat and KE-37 (both CD74 negative) were treated with various concentrations of the CD74-targeting ADC STRO-001 (red lines) or, as a control, an isotype-matched ADC recognizing GFP (GFP-SC236; blue lines), as indicated. After 72 h, induction of cell death was determined by PI staining and flow cytometry. The percentage of viable cells is indicated. Right, indication of the LD_50_ for STRO-001 for the various cell lines. Note that below 5 μg/mL, specifically ALCL cells are killed by ADC-STRO-001, and that the control ADC GFP-SC236 does not exert cytotoxicity on any of the cell lines. One out of three independent experiments is shown. (**B**) K299, Mac-2A, FE-PD and Jurkat cells were left untreated (0 h), or treated for 24 and 48 h with STRO-001 or, as a control, GFP-SC236. At the indicated times, whole cell extracts were prepared and analyzed by immunoblotting using antibody specifically recognizing cleaved PARP-1 (Asp214) (left panels) or antibody recognizing full-length PARP-1 and its large cleavage product (the latter marked by *; right panels). Note that an increase in cleaved PARP-1 is only detected in ALCL cell lines treated with STRO-001. One out of three independent experiments is shown. (**C**–**E**) K299 (**C**), FE-PD (**D**) and Jurkat (**E**) cells were left untreated (control, left) or treated for 48 h with STRO-001 (right) or, as a control, GFP-SC236 (center). Thereafter, cells were analyzed by Annexin V-FITC/PI-staining for induction of apoptotic cells. The percentages of cells in the respective quadrants are indicated. Note that an increase in Annexin V-FITC-positive cells is only detectable in ALCL cell lines treated with STRO-001. One out of three independent experiments is shown.

Given the robust expression of CD74 on ALCL, and the rapid internalization of CD74, we reasoned that targeting CD74 by specific antibody-drug conjugates might be a possible treatment strategy for ALCL. To this end, we treated various ALCL and non-ALCL cell lines in vitro with the recently developed antibody-drug conjugate STRO-001, which is composed of an aglycosylated anti-CD74 IgG1 human antibody conjugated to a non-cleavable linker-maytansinoid warhead [42] (Figure 5A and Figure S3). Cells were treated with STRO-001 or, as a control, an isotype-matched ADC targeting GFP (GFP-SC236). As determined after 72 h, at concentrations below 5 μg/mL STRO-001 efficiently induced cell death of all the CD74-postive ALCL cell lines K299, JB6, FE-PD and Mac-2A, whereas no similar cell death induction was observed in the non-ALCL T cell control cell lines Jurkat and KE-37. The isotype-matched control ADC GFP-SC236 did not affect viability of any of the cell lines (Figure 5A and Figure S3). Furthermore, we analyzed whole cell extracts following treatment with STRO-001 or GFP-SC236 by immunoblotting for PARP-1 cleavage using antibody specific to cleaved PARP Asp214 (Figure 5B, left panels; Figure S3) as well as antibody recognizing both full-length PARP and PARP cleavage products (Figure 5B, right panels; Figure S3). These analyses in K299, Mac-2A and FE-PD cells revealed PARP-1 cleavage by STRO-001 but not by ADC GFP-SC236 or in CD74-negative Jurkat cells, suggesting specific induction of apoptosis by STRO-001 in the ALCL cell lines. Induction of apoptotic cell death was in addition demonstrated by the increase in Annexin V-positive K299 (Figure 5C and Figure S3) and FE-PD (Figure 5D and Figure S3) cells following STRO-001 treatment, but was not observed following treatment with the control ADC or treatment of CD74-negative Jurkat cells (Figure 5E and Figure S3).

## 4. Discussion

Based on our previously developed pathogenic concept that ALK^+^ and ALK^−^ ALCL share a common molecular basis of transformation [14,17,18], we report here the identification of deregulated CD74 expression as a further candidate gene in support of this view. Since its initial discovery, the expression of CD74 has been studied particularly in various B cell compartments as well as B cell- and epithelial cell-derived malignancies [19,39]. Among hematopoietic cells, these analyses identified CD74 expression as a unifying feature of antigen-presenting cells or their malignant counterparts, being most prominent in cells of B cell origin. In contrast, except for subpopulations after activation, T cells usually lack CD74 expression [21,27,49]. CD74 expression in T cell-derived malignancies has been previously reported for one Sézary syndrome cell line [24], and only recently has CD74 been analyzed in various lymphoma subtypes including T cell-derived malignancies [28]. Remarkably, apart from B-NHL of various subtypes, ALCL showed the most consistent expression pattern. We here provide further evidence that CD74 expression is a common feature of T cell-derived ALK^+^ and ALK^−^ ALCL, which might at least in part be due to an altered DNA methylation pattern of the *CD74* gene locus in ALCL. In a previous report on pediatric ALCL, CD74 staining could not be detected on ALCL cells [50]. The reason for that might primarily be methodical differences; a less possible explanation is that pediatric ALCL, which in most cases is ALK^+^, differs from adult ALK^+^ ALCL with respect to CD74 expression.

Effects of CD74 engagement have been intensively studied in B cells, demonstrating diverse consequences such as induction of cellular proliferation, survival or cell death depending on the cell type and differentiation stage [19,34,35,51]. In a number of B cell-derived malignancies, preclinical models confirmed the efficacy of anti-CD74 targeting as a therapeutic principle [39,42]. Furthermore, combinatory treatment approaches using anti-CD74 together with anti-CD20 antibodies resulted in enhanced induction of cell death as shown for mantle cell lymphoma (MCL) cells [51]. Therefore, anti-CD74 antibodies are currently being tested in clinical trials for the treatment of patients with B cell malignancies [39,52] and might represent a broad treatment option for these disease entities. In contrast, for T cell-derived malignancies, antigens with similarly broad expressed patterns suitable for targeted treatment strategies are rare. Only recently has the approval of armed anti-CD30-targeting antibodies for the treatment of ALCL patients [53,54] demonstrated the power of immunotherapeutic approaches also for these malignancies. The consistent expression of CD74 in ALCL in combination with our functional data suggest that targeting CD74 should be further explored in T cell malignancies. Thus, CD74 belongs to the few druggable targets shared between cells of B and T cell origin, and it has to be determined whether preclinical in vitro and in vivo data as well as results from clinical studies obtained in B-NHL can be transferred to T cell malignancies. Whether targeting of CD74 might act synergistically together with CD30-targeting antibodies such as brentuximab vedotin has to be investigated in future studies.

We can only speculate about the biological function of the aberrantly expressed CD74 (and expression of CTIIA and MHC-II family members at least in ALCL cell lines) in ALCL. Obviously, in vivo, CD74 expression in ALCL does not result in cell death as observed following enforced antibody-mediated ligation. The biological function of aberrant CD74 expression and associated signaling in ALCL might be the substitution for TCR-mediated signaling, since most components of the T cell receptor complex are down-regulated to some extent in ALCL [12,14]. In fact, substitution of TCR signaling might be required at least at some point of ALCL pathogenesis [55]. In this context, it is interesting to note that ALCL regularly develops at sites of chronic, possibly non-specific antigen stimulation, for example, breast implants or insect bites [56,57], suggesting a link between chronic T cell stimulation and ALCL pathogenesis. Even more intriguing, aberrant expression or maintenance of CD74 and its fragment CLIP on solid tumor cells and lymphoma models prevents presentation of tumor antigens and favors an immune escape [58,59,60], which could contribute to prevention of an effective anti-lymphoma immune response in ALCL. Apart from effects on cell growth and survival, the aberrant expression of MHC-II-associated regulators such as CTIIA alters T cell polarization [61], which might contribute to the ALCL phenotype. Furthermore, the influence of CD74 on NF-κB or AP-1 activation [30,35] in ALCL needs to be determined in future studies. As an additional tool for possible therapeutic interventions in ALCL, we demonstrated in this study MET expression in a subset of cases. MET is known to form signaling complexes with CD74 [33,46], and can be activated by CD74 engagement [62]. Although we did not observe an effect of MET inhibitors on ALCL cell viability, the functional role of MET, alone or in the context of CD74 signaling, in ALK^+^ ALCL has to be investigated in future studies.

Taken together, our data extend the spectrum of CD74-positive diseases to ALK^+^ and ALK^−^ ALCL. Given the usually limited prognosis of subgroups of these lymphoma entities and of relapsed and refractory disease, CD74 and its associated signaling components represent promising candidates for the development of new therapeutic strategies for these T cell-derived malignancies.

## 5. Conclusions

In conclusion, our findings support the concept that ALK^+^ and ALK^−^ ALCL share a common molecular basis of transformation. More specifically, we describe robust expression of CD74, also known as the invariant chain of major histocompatibility complex [MHC]-II, in ALCL regardless of t(2;5). The fact that CD74 ligation induces cell death of ALCL cells and enhances the cytotoxic effects of chemotherapeutics or the ALK inhibitor crizotinib in ALCL cell lines, as well as the efficient killing of CD74-positive ALCL cell lines by a CD74-targeting antibody-drug conjugate, all point to the exploration of CD74 as a therapeutic tool for ALCL. In addition, we demonstrated MET-expression in a subset of ALCL cases. Together, these data provide new insights into ALCL biology and might help to identify new targets for the development of treatment strategies for this lymphoma type.

## Figures and Tables

**Table 1:** Staining in ALK^+^ and ALK^−^ ALCL.

Lymphoma Entity	No. of Cases		IHC Staining Intensity				
		Absent	+	+/++	++	++/+++	+++
ALCL ALK^+^	35	0	8	15	11	1	0
ALCL ALK^−^	21	0	3	8	5	4	1

The percentage of positive ALCL cells in each case was unrelated to the staining intensity and was as follows: among the ALK^+^ ALCL cases, 100% of the lymphoma cells stained positive in 14, 70–90% in another 14, 50–70% in 3, and <50% in 4; among ALK^−^ ALCL cases, 100% of the lymphoma cells stained positive in 13, 70–90% in 5, and <50% in 3. Staining intensities are indicated as follows: +, weak; ++, intermediate; +++, strong.

## Data Availability

All data supporting the findings of this study are available within the paper and its supplementary information files. All DNA methylome data produced in this study will be deposited in publicly accessible databases.

## Supplementary Materials

The following are available online at https://www.mdpi.com/article/10.3390/cancers13195012/s1, Figure S1: Analysis of CD74 expression in various cell lines; control stainings for CD4, CD19 and CD74 double staining analyses on human PMNC; time course of apoptosis induction by CD74 ligation; analyses of *CD44*, *CXCR4* and *MIF* mRNA expression in various cell lines, Figure S2: Blots used for presentation of specific bands in Figure 1A, Figure 4A and Figure S1A, Figure S3: Blots used for presentation of specific bands in Figure 5. Supplementary Material containing citations for publications used for analyses of array-based DNA methylation data.
