# Supplementary material for: Aberrant Expression of and Cell Death Induction by Engagement of the MHC-II Chaperone CD74 in Anaplastic Large Cell Lymphoma (ALCL)
Source: Cancers (Basel). 2021 Oct 7;13(19):5012. doi: 10.3390/cancers13195012 (PMC8507667; doi:10.3390/cancers13195012)
Supplement: Supplementary file 1 [file cancers-13-05012-s001.zip › cancers-1400211-supplementary.pdf]

Supplementary Figure 1

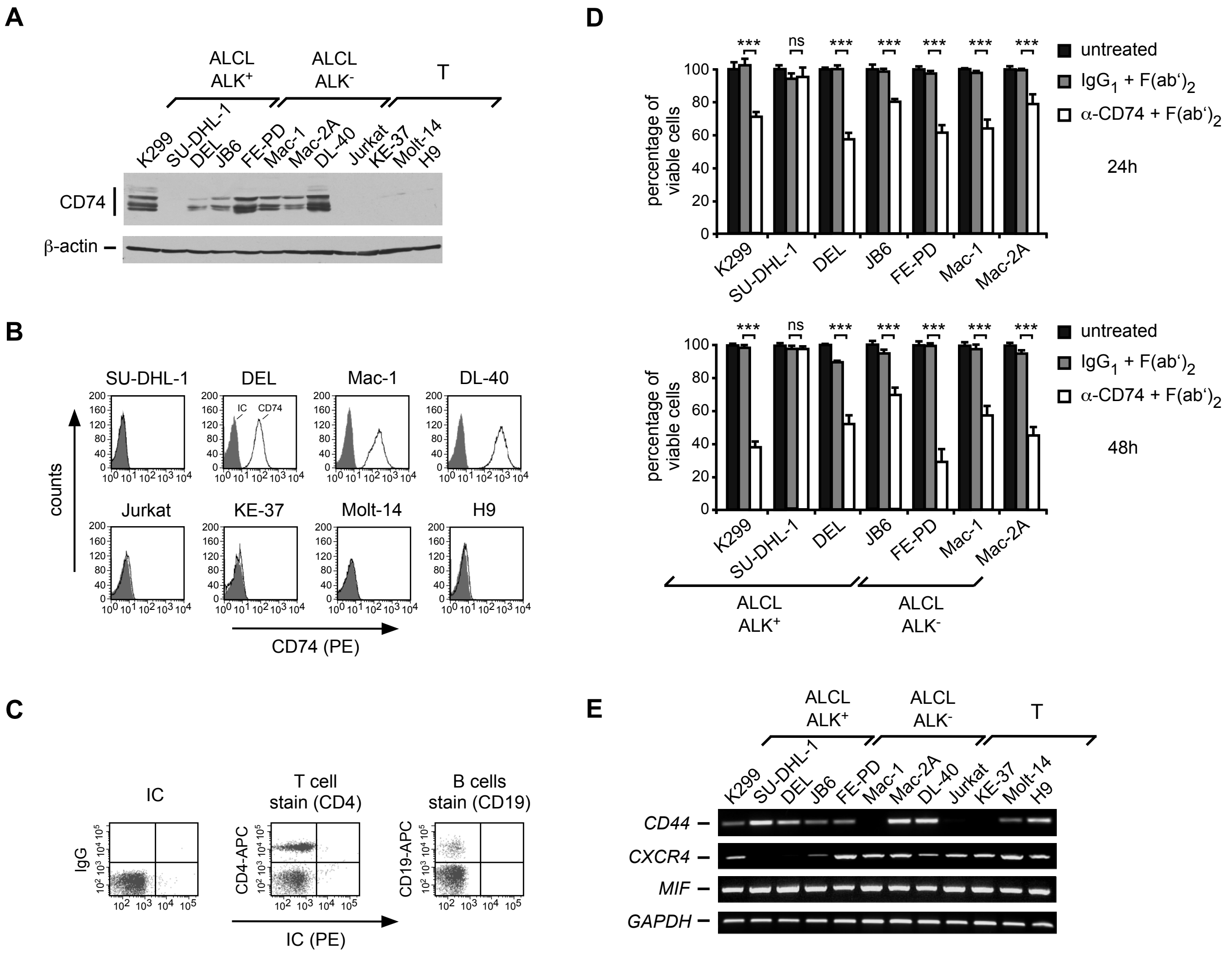

Supplementary Figure 1. (**A**) Analysis of CD74 protein expression by use of antibody sc-20062 in various ALK$^+$ and ALK$^-$ ALCL as well as T cell control cell lines, as indicated. The expression of β-actin was analyzed as a control. Note, that various CD74 protein bands of different sizes are detectable. (**B**) Cell surface expression analysis of CD74 in various ALCL (SU-DHL-1, DEL, Mac-1, DL-40) and T cell control (Jurkat, KE-37, Molt-14, H9) cell lines by extracellular flow cytometry (antibody sc-20062). Open histogram, CD74 staining; filled histogram, isotype control (IC). (**C**) Control stainings for CD4 or CD19 and CD74 double staining analyses of human PMNC by extracellular flow cytometry. Note, that the isotype control (IC) antibody does not result in non-specific staining. (**D**) Time course of apoptosis induction by CD74 ligation in various ALCL cell lines. Various ALK$^+$ and ALK$^-$ cell lines were left untreated (black columns), or were treated with 5 µg anti-CD74 in combination with 20 µg F(ab')$_2$ (open columns), or anti-IgG$_1$ in combination with F(ab')$_2$ (gray columns). Note, that SU-DHL-1 cells, which lack CD74 expression, do not respond to CD74 ligation. One of four independent experiments is shown. Error bars denote SDs. ***, $P < 0.0001$. ns, not significant. (**E**) Analysis of *CD44*, *CXCR4* and *MIF* mRNA expression by semiquantitative RT-PCR in various ALK$^+$ and ALK$^-$ ALCL as well as T cell control cell lines, as indicated. The expression of *GAPDH* was analyzed as a control.

Supplementary Figure 2

Blots used in Figure 1A

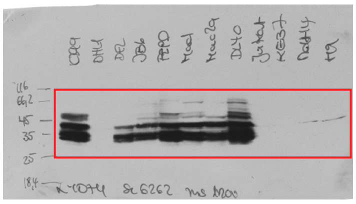
CD74

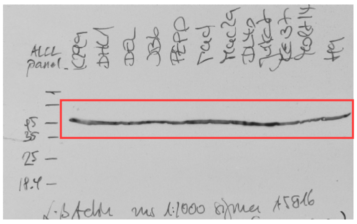
β-actin

Blots used in Supplementary Figure 1A

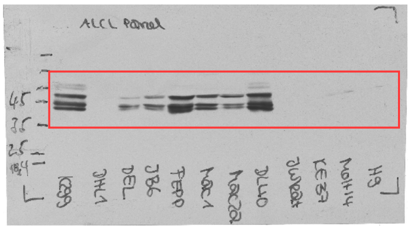
CD74

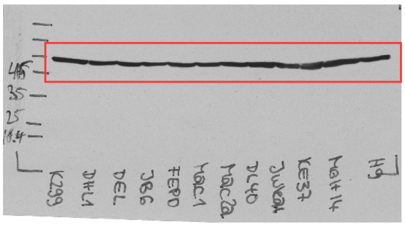
β-actin

Blots used in Figure 4A

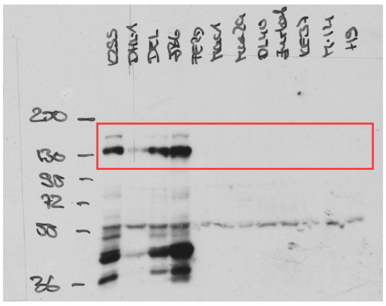
MET

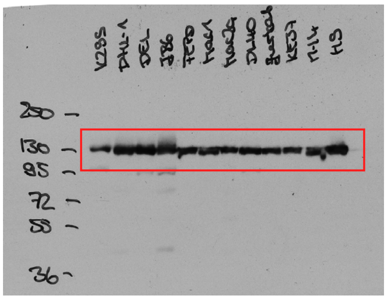
PARP

Supplementary Figure 2. Blots used for presentation of specific bands in Figure 1A, Figure 4A and Supplementary Figure 1A, as indicated. Bands shown in the respective figure parts are marked by red rectangles.

Supplementary Figure 3

Blots used in Figure 5B, left

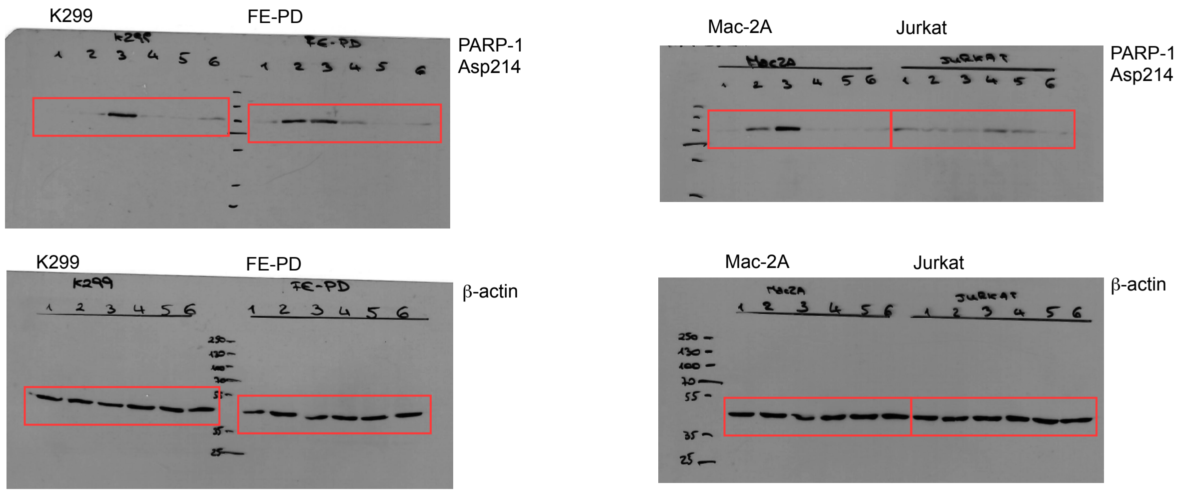

Blots used in Figure 5B, right

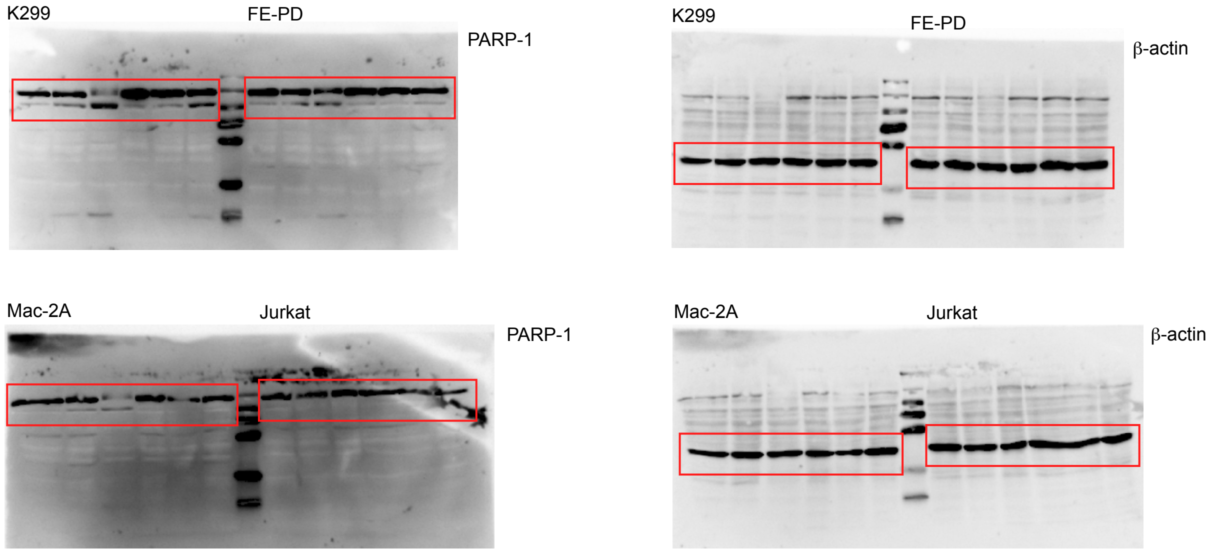

Supplementary Figure 3. Blots used for presentation of specific bands in Figure 5. Bands shown in the respective figure parts are marked by red rectangles.

Supplementary Material; Wurster *et al*., Aberrant expression of and cell death induction by engagement of the MHC-II chaperone CD74 in anaplastic large cell lymphoma (ALCL)

DNA methylation analyses using Illumina Infinium Arrays

For the DNA methylation analyses, besides neoplastic samples the study cohort was complemented with publically available data from various cell populations covering T- and B-lineage differentiation as well as from monocytes and macrophages from the following publications:

1. Bergmann AK, Fataccioli V, Castellano G, *et al*. DNA methylation profiling of hepatosplenic T-cell lymphoma. *Haematologica*. 2019;**104**:e104-e107.

2. Garci-Gomez A, Li T, Kerick M, *et al*. TET2- and TCG-mediated changes are required for the acquistition of distinct histone modifications in divergent terminal differentiation of myleoid cells. *Nucleic Acids Research*. 2017;**45**:10002-10017.

3. Hassler MR, Pulverer W, Lakshminarasimhan R, *et al*. Insights into the pathogenesis of anaplastic large-cell lymphoma through genome-wide DNA methylation profiling. *Cell Rep*. 2016;**17**: 596-608.

4. Husquin LT, Rotival M, Fagny M, *et al*. Exploring the genetic basis of human population differences in DNA methylation and their causal impact on immunce gene regulation. *Genome Biol*. 2018;**19**:222.

5. Kennedy JJ, Goehring GN, Nichols MH, *et al*. An integrated-omics analysis of the epigenetic landscape of gene expression in human blood cells. *BMC Genomics*. 2018;**19**:476.

6. Kretzmer H, Bernhart SH, Wang W, *et al*. DNA methylome analysis in Burkitt and follicular lymphomas identifies differentially methylated regions linked to somatic mutation and transcriptional control. *Nat. Genet*. 2015;**47**:1316–1325.

7. Kulis M, Merkel A, Heath S, *et al*. Whole-genome fingerprint of the DNA methylome during human B cell differentiation. *Nat. Genet*. 2015;**47**:746–756.

8. Lee S-T, Xiao Y, Muench MO, *et al*. A global DNA methylation and gene expression analysis of early human B-cell development reveals a demethylation signature and transcription factor network. *Nucleic Acids Res*. 2012;**40**:11339–11351.

9. Oakes CC, Seifert M, Assenov Y, *et al*. DNA methylation dynamics during B cell maturation underlie a continuum of disease phenotypes in chronic lymphocytic leukemia. *Nat. Genet*. 2016;**48**:253-264.

10. Rodriguez RM, Suarez-Alvarez B, Mosén-Ansorena D, *et al*. Regulation of the transcriptional program by DNA methylation during human αβ T-cell development. *Nucleic Acids Res*. 2015;**43**:760–774.

11. Vento-Tormo R, Company C, Rodriguez-Ubreva J, *et al*. IL-4 orchestrates STAT6-mediated DNA demethylation leading to dendritic cell differentiation. *Genome Biology*. 2016;**17**, 4.
